# A Kinematic Study of Progressive Micrographia in Parkinson's Disease

**DOI:** 10.3389/fneur.2019.00403

**Published:** 2019-04-24

**Authors:** Poonam Zham, Sanjay Raghav, Peter Kempster, Sridhar Poosapadi Arjunan, Kit Wong, Kanae J. Nagao, Dinesh K. Kumar

**Affiliations:** ^1^School of Engineering, RMIT University, Melbourne, VIC, Australia; ^2^Department of Neurosciences, Monash Medical Centre, Clayton, VIC, Australia; ^3^Department of Medicine, Monash University, Clayton, VIC, Australia

**Keywords:** Parkinson's disease, handwriting, kinematic, progressive micrographia, bradykinesia

## Abstract

Progressive micrographia is decrement in character size during writing and is commonly associated with Parkinson's disease (PD). This study has investigated the kinematic features of progressive micrographia during a repetitive writing task. Twenty-four PD patients with duration since diagnosis of <10 years and 24 age-matched controls wrote the letter “*e*” repeatedly. PD patients were studied in defined *off* states, with scoring of motor function on the Unified Parkinson's Disease Rating Scale Part III. A digital tablet captured *x-y* coordinates and ink-pen pressure. Customized software recorded the data and offline analysis derived the kinematic features of pen-tip movement. The average size of the first and the last five letters were compared, with progressive micrographia defined as >10% decrement in letter stroke length. The relationships between dimensional and kinematic features for the control subjects and for PD patients with and without progressive micrographia were studied. Differences between the initial and last letter repetitions within each group were assessed by Wilcoxon signed-rank test, and the Kruskal-Wallis test was applied to compare the three groups. There are five main conclusions from our findings: (i) 66% of PD patients who participated in this study exhibited progressive micrographia; (ii) handwriting kinematic features for all PD patients was significantly lower than controls (*p* < 0.05); (iii) patients with progressive micrographia lose the normal augmentation of writing speed and acceleration in the *x* axis with left-to-right writing and show decrement of pen-tip pressure (*p* = 0.034); (iv) kinematic and pen-tip pressure profiles suggest that progressive micrographia in PD reflects poorly sustained net force; and (v) although progressive micrographia resembles the sequence effect of general bradykinesia, we did not find a significant correlation with overall motor disability, nor with the aggregate UPDRS-III bradykinesia scores for the dominant arm.

## Introduction

Micrographia is common in Parkinson's disease (PD) and may predate other symptoms (1, 2). Kinnear Wilson (3), had proposed a subdivision into consistent micrographia, where the size of letters is reduced by the same degree over multiple repetitions, and progressive micrographia (PMG), with decrement of letter size. While only a minority of subsequent publications have emphasized this distinction (4), one recent study suggested that the two types of parkinsonian micrographia show different patterns of activation of the motor system on functional MRI scans (5).

Elements of bradykinesia—slowness, reduced range of movement, loss of rhythmicity, and decrement of repeated action—appear to contribute to handwriting difficulty in PD. Yet this relationship is not straightforward, and micrographia can be present in the absence of detectable bradykinesia (6). The motor decrement of typical bradykinesia may be analogous to the decrement of PMG. Consistent micrographia, on the other hand, suggests purer hypokinesia, such as is sometimes seen in progressive supranuclear palsy (7). Strictly speaking, consistent micrographia requires inspection of pre-morbid calligraphy to ascertain the reduction in script size. To overcome this limitation, Kim et al. (8) proposed a method based on comparison with the mean size of writing obtained from age- and sex-matched control subjects. Classified in this way, some PD patients are found to have both consistent and progressive writing deficits (5).

Computerized graphics tablets allow investigation of the dimensional and kinematic features of handwriting as well as pen pressure. This technology can identify PD patients at an early stage of the disease course and can monitor its progression (9, 10). It has been shown that stroke size, velocity, and peak acceleration are impaired in PD (11, 12), and that kinematic features are more sensitive than size for detecting early PD (13).

It has also been shown that progressive micrographia varies according to writing task (4, 8, 14). One study of successive writing strokes by computerized methods did not find any change in size but saw an increase in stroke duration in PD (15). Using a more advanced digital tablet, Van Gemmert et al. (11) found that stroke size decreases while the stroke duration remains unchanged.

The definition of consistent micrographia being somewhat problematic, we chose instead to focus on the presence or absence of PMG. In a departure from previous studies that relied on the standard deviation of control letter size to determine PMG, we opted for an absolute definition. We chose a 10% decrement, based on the smallest change in the handwriting that could be easily discerned by eye, and respecting Kinnear Wilson's principle that micrographia is “an obvious reduction in the size of letters” (3). Earlier studies have shown that there is significant variability in the size of free-flow handwriting of healthy people (16). This variability is dependent on number of factors such as age, level of education and mother tongue. An inherent shortcoming in the use of standard deviation of control participants to identify PMG in PD is a lack of comparability from study to study. While this is less significant when language skills and demographics of participants are similar, the limitation increases with a multicultural cohort, especially when making comparisons across writing cultures. By means of a computerized study of pen movements in PD subjects, we investigated the kinematic features of PMG, and the extent to which it mirrors parkinsonian bradykinesia and its motor decrement phenomenon.

The premise of this study was that PMG is an important aspect of parkinsonian dysgraphia, and that kinematic findings should distinguish PD patients with and without this writing deficit. Furthermore, we hypothesized that PMG and the motor decrement of parkinsonian bradykinesia are closely related motor phenomena.

## Materials and Methods

### Participants

Twenty-four patients diagnosed with PD within the last 10 years were recruited from the Movement Disorders Clinic at Monash Medical Center. All complied with the Queen Square Brain Bank criteria for idiopathic PD (17). Presence of any advanced disease clinical milestone—visual hallucinations, frequent falling, cognitive disability, need for institutional care—was an exclusion criterion (18). Motor function was scored by a neurologist in a practically defined *off* state (anti-parkinsonian medication withheld for at least 12 h) on the Unified Parkinson's Disease Rating Scale Part III (UPDRS-III) (19). Dominant upper limb subscores for finger tapping, hand movements and pronation-supination [UPDRS sections 3.4–3.6] gauged the amount of bradykinesia in the writing hand. Twenty-four healthy age-matched controls were recruited from various retirement villages. Demographic details of all participants are as shown in Table 1. The study was conducted in accordance with the Helsinki Declaration on human experiments (revised 2004) and approved by the Monash Health and RMIT University Human Research Ethics Committees. All participants in this study gave their written informed consent prior to data recording.

**Table 1:** Demographic and clinical information, PD patients and controls.

	**PD**	**Control group**	***p*-value**
Number of subjects	24	24	
Age, years	71.6 ± 7.14	69.3 ± 5.74	0.2^a^
Gender (male, female)	13,11	14,10	1.0^b^
Hand dominance for writing (right, left)	20,4	22,2	0.7^b^
Disease duration, years	5 ± 2.88	–	
UPDRS-III OFF [0–132]	26.80 ± 9.50	–	
UPDRS-III dominant upper limb bradykinesia score [0–12]	3.58 ± 1.64	–	

Values are mean ± SD, Comparison between groups is performed using

a independent t-test and

b *2-tailed Chi-Square test*.

### Experimental Methods

A digital tablet (Wacom Intuos Pro-Large) was used for the experiments. The tablet captured *x*-*y* coordinates and ink-pen pressure on its surface at a sampling rate of 133 Hz, which was time-stamped. This device was selected on the basis of feedback from participants in a previous study, who preferred its A3 size and the feeling of conventional pen and paper. The letters were written on paper, which was attached to the tablet. The position of the tablet was adjusted for each participant, who was seated in front of an adjustable desk. Customized software was used to record the data from the tablet and perform off-line analysis.

### Handwriting Tasks

**Figure 1:**
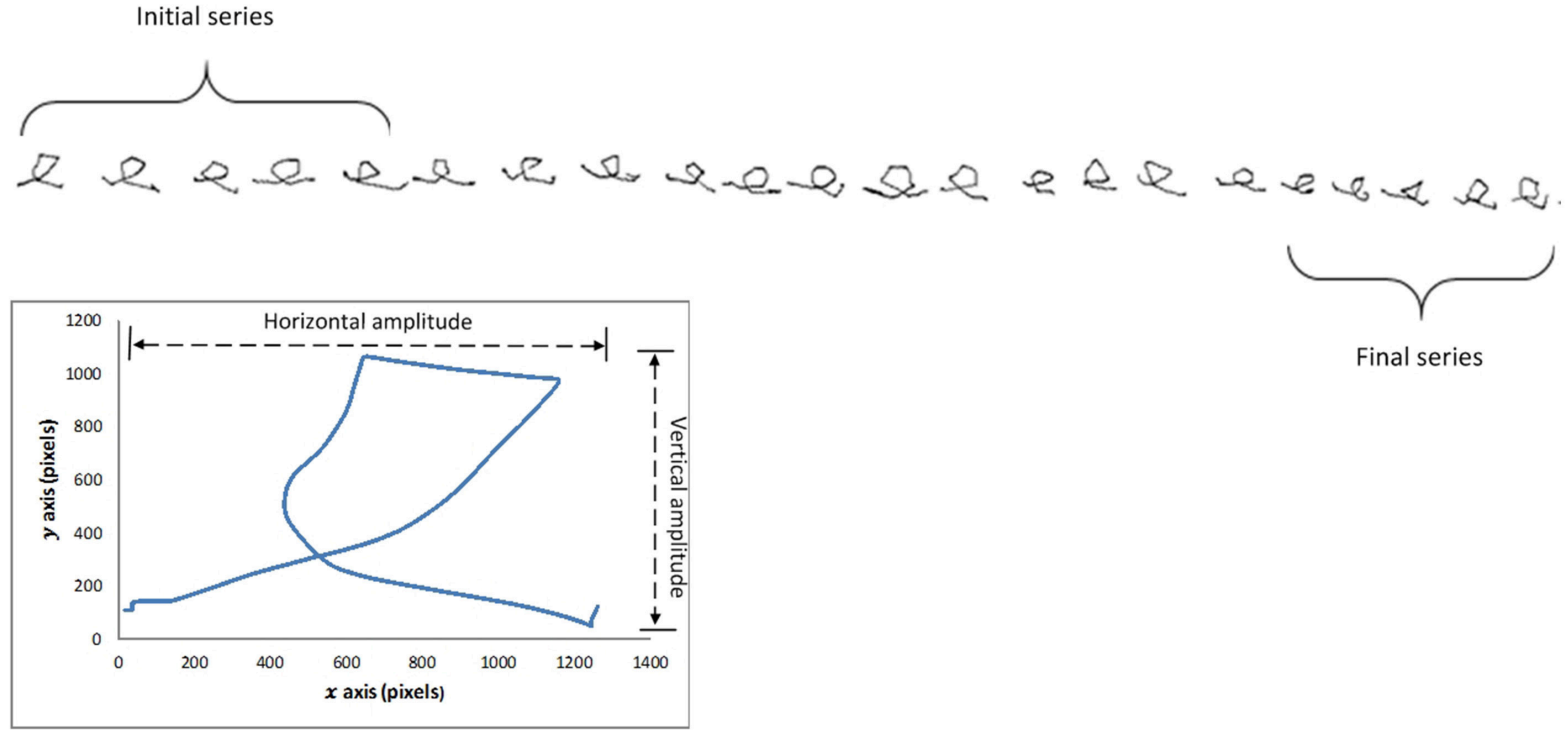
Letter e sequence from subject with progressive micrographia, showing selection of first and last five letters. Enlarged single letter illustrates the relationship between stroke length and horizontal and vertical amplitudes.

Participants were instructed to write the letter *e* repeatedly, with pen-up at the end of each letter (see Figure 1). Once 20 repetitions had been exceeded, a researcher gave the instruction to stop writing. Similar protocols have previously been used to study micrographia (8, 15, 20).

### Computation of Parameters

The writing data consisted of four columns corresponding to time-stamp (*t*), *x, y* and pen-tip pressure (*p*). This was first segmented to identify individual letters based on pen-up and pen-down obtained from the pen-tip pressure data. Segments of length < 5 mm were found to be noise and were disregarded. The results were inspected to confirm the segmentation.

We computed character size by two methods. Quadrilateral letter area had been employed in previous studies of Chinese characters (5, 14). Because of the difference in the two scripts, Chinese characters having a square shape consisting of multiple pen strokes whereas the Roman alphabet character *e* has a rounded form of a single stroke, we calculated the stroke length of each character (*S*_*i*_) as our primary measure of character size (21). Stroke length was based on Euclidean distance where *m* indicates number of points obtained from the time when pen touches the surface till it leaves the surface and *i* is the total number of characters (Figure 1):

$$S_{i}=\sum_{n=0}^{m}\sqrt{{\left(x_{n}-x_{n-1}\right)}^{2}+{\left(y_{n}-y_{n-1}\right)}^{2}}$$

The first and last set of 5 *e* characters written by each participant were compared (see Figure 1). PD subjects showing >10% reduction in average letter stroke length were labeled as PD_pmg, the others as PD_o. Measuring the relative change in handwriting size of the individual ensured that inter-participant variations do not affect the results. Consistent micrographia was defined as mean letter size below two standard deviations of controls, as proposed by Kim et al. (8).

The selection of the kinematic features was based on previously published work. In addition to speed and pen-tip pressure, acceleration in *x* and *y* directions were computed (22, 23). A pilot study was conducted and it was observed that the pen-tip pressure settled in <3 samples, or corresponding to <5%. As pen-tip pressure recorded by the Wacom digital tablet is unitless, we calibrated the device to obtain equivalent forces in Newton (N). Normalized pen-tip pressure for each participant was calculated using the formula (*P*_*Avg*_ − *P*_min_)/(*P*_max_ − *P*_min_), where *P*_*max*_ and *P*_*min*_ are the highest and lowest pen-tip pressures recorded across all participants, and *P*_*Avg*_ is an individual's average pressure.

Linear correlation was observed between weight and pen-tip pressure levels (24).

The full list of features is listed in Table 2. For each feature, the mean values of the initial and final sets of 5 *e* characters was obtained.

**Table 2:** Features calculated for first and last e series.

**Feature**	**Feature description**
Stroke length *S*	Length of continuous pen stroke to produce letter e.
Quadrilateral area	Area of the quadrilateral outlined by the upper, lower, left and right margins of each letter.
Horizontal amplitude	See Figure 1.
Vertical amplitude	See Figure 1.
Speed	The speed of the pen tip while moving on the surface.
Normalized Pen-tip Pressure	Calibrated and calculated as detailed in Materials and Methods.
Acceleration in *x* direction	The rate of change of velocity of the pen tip in the x-direction.
Acceleration in *y* direction	The rate of change of velocity of the pen tip in the y-direction.

### Statistical Analysis

Independent sample *t*-test, 2-tailed Chi-Square test and Mann Whitney *U* test were performed to compare various demographic features. Based on the Shapiro-Wilk test, non-parametric Wilcoxon signed rank test was performed to analyse the difference between initial and final values for size and other kinematic feature for each group separately. The three groups were compared using distribution-free Kruskal-Wallis with the *post-hoc* test (25). Spearman's rank correlation coefficient analysis was performed to study the relationship between UPDRS-III scores and kinematic features.

In designing this research, the sample size of 24 in each group was determined by power calculation performed using online power and sample size calculator (26). This was based on the statistical power of 0.8 with 95% confidence interval, with the null hypothesis being the existence of mean difference between the groups.

## Results

Sixteen out of 24 PD subjects were classified as PD_pmg by a 10% reduction of stroke length between first and final letters. Four control subjects also met the definition for PMG, though their kinematic measures showed little difference, of no statistical significance, from the remainder of the control group. Statistical analysis of demographic features showed no significant differences between PD_pmg and PD_o groups (Table 3).

**Table 3:** Demographics of PD_pmg and PD_o groups.

	**PD_pmg**	**PD_o**	***p*-value**
Number of subjects	16	8	
Age, years	70.94 ± 7.59	73.63 ± 6.23	0.4^a^
Gender (male, female)	10,6	3,5	0.35^b^
Handedness (right, left)	13,3	7,1	0.83^b^
Disease duration, years	5.1 ± 2.8	5.3 ± 3.2	0.84^a^
UPDRS-III *OFF* [0–132]	28.5 ± 10.33	23.88 ± 7.86	0.28^a^
UPDRS-III dominant upper limb bradykinesia score [0–12]	3.56 ± 1.79	3.75 ± 1.28	0.79^a^

Values shown as mean ± SD. Comparisons between groups performed using

a independent t-test and

b *Mann-Whitney U test*.

By stroke length, 4 out of 24 PD participants were found to have consistent micrographia, though 3 out of these also showed PMG, leaving only one case of pure consistent micrographia. Using the quadrilateral letter area method, none of the participants showed consistent micrographia.

Table 4 shows the median values, effect size *r*, and *p*-values of the size, area, horizontal and vertical amplitude, pen-tip pressure, and kinematic features for paired initial and final 5 repetitions of the character *e*. Table 5 shows a summary of the trends observed in Table 4. Letter area as well as stroke length showed decrement from initial to final *e* series in the PD_pmg group with large effect size (*r* = 0.62) (27, 28). There was a reduction of the vertical amplitude for all the 3 groups (*p* < 0.05) over the duration of the task, this effect was most significant (*p* < 0.001) in PD subjects with PMG. Median horizontal amplitude was preserved in PD and actually increased in controls. The PD_o and control groups show a significant increment (*p* < 0.05) from initial to the final series for pen speed and acceleration in x-direction with moderate to large effect size. The PD_pmg group, however, showed no significant differences across the task for these kinematic features. While pen-tip pressure did not change significantly for the PD_o and control groups, the PD_pmg group was unable to maintain pen pressure through the exercise.

**Table 4:** Kinematic and dimensional features of handwriting of PD and control groups, presented with group median, effect size and *p*-values from *exact* 2-tailed Wilcoxon signed rank test.

**Series**	**PD_pmg**			**PD_o**			**Controls**		
	**Median**	**Effect Size (r)**	***P***	**Median**	**Effect Size**	***P***	**Median**	**Effect Size (r)**	***P***
**STROKE LENGTH (mm)**									
Initial	18.97	0.62	< 0.001	16.35	0.53	0.039	19.4	0.22	0.128
Final	14.61			17.63			19.44		
**QUADRILATERAL AREA(mm**^**2**^**)**									
Initial	30.11	0.63	< 0.001	28.35	0.35	0.2	30.39	0.14	0.36
Final	18.41			25.41			26.77		
**HORIZONTAL AMPLITUDE (mm)**									
Initial	5.31	0.10	0.6	5.75	0.25	0.38	6.16	0.47	< 0.001
Final	5.76			6.91			7.39		
**VERTICAL AMPLITUDE (mm)**									
Initial	4.24	0.62	< 0.001	5.81	0.53	0.04	4.53	0.33	0.023
Final	3.62			4.34			3.71		
**SPEED (mm/s)**									
Initial	20.70	0.1	0.98	19.03	0.63	0.008	38.76	0.45	0.001
Final	20.68			26.67			41.23		
**PEN-TIP PRESSURE NORMALIZED:0–1 (NEWTON N)**									
Initial	0.474 (0.22 N)	0.37	0.034	0.493 (0.23N)	0.04	0.945	0.55 (0.25N)	0.04	0.79
Final	0.408 (0.19 N)			0.472 (0.22N)			0.52 (0.24N)		
**ACCELERATION IN X DIRECTION (mm/sec**^**2**^**)**									
Initial	313.453	0.23	0.211	318.26	0.6	0.016	749.2	0.52	0.008
Final	392.885			494.31			999.65		
**ACCELERATION IN Y DIRECTION (mm/sec**^**2**^**)**									
Initial	206.57	0.12	0.562	222.22	0.07	0.844	530.85	0.09	0.55
Final	235.25			260.5			530.11		

**Table 5:** Group trends, initial vs. final characters.

**Group**	**Amplitude**		**Stroke length**	**Area**	**Speed**	**Pen-tip pressure**	**Acceleration**	
	**Horizontal**	**Vertical**					**Horizontal**	**Vertical**
PD_pmg	No change	Decrease	Decrease	Decrease	No change	Decrease	No change	No change
PD_o	No change	Decrease	Increase	No change	Increase	No change	Increase	No change
Controls	Increase	Decrease	No change	No change	Increase	No change	Increase	No change

To test the difference between the 3 group independent samples, Kruskal-Wallis with the *post-hoc* test was performed for the series. While there was no significant difference between PD_pmg and PD_o, PD_pmg and controls showed a significant difference (*p* < 0.05, adjusted using the Bonferroni correction) for all kinematic features except pen-tip pressure (*p* > 0.5). PD_o and controls showed a significant difference for all the features except Speed *s* (*p* = 0.064). Spearman rho values did not reveal any significant correlation between UPDRS-III scores and kinematic features in PD subjects.

## Discussion

Handwriting is a learned motor skill, which requires coordinated movement of fingers, wrist and arm. It can be impaired at an early stage of PD, and is a good model from which to analyse the effects of basal ganglia disease on the planning and execution of habitual actions. In cursive handwriting, the primary role of the thumb, index and middle fingers is in vertical penstrokes, while wrist flexion and extension generates small lateral movements (2). As handwriting progresses from left to right across a writing surface, the involvement of the wrist and elbow increases (22). These different patterns of muscle activation produce progressive changes in normal linear writing. Our control group maintained overall size and area of letters; horizontal amplitude increased across a line while vertical amplitude, possibly because of fatigue of smaller muscles controlling finger movement, decreased. The speed of writing increased in the horizontal but not in the vertical direction (29). We did not see any significant kinematic changes from first to final letter series in the vertical direction for any of the groups. The differences lay in the horizontal direction. Both the control and PD_o subjects showed an increase in writing speed and acceleration in the *x* axis. This probably reflects changes in muscle activation as wrist and elbow movement come increasingly into play when writing from left to right. These increases are not present in the 67% of PD patients who exhibited PMG.

**Figure 2:**
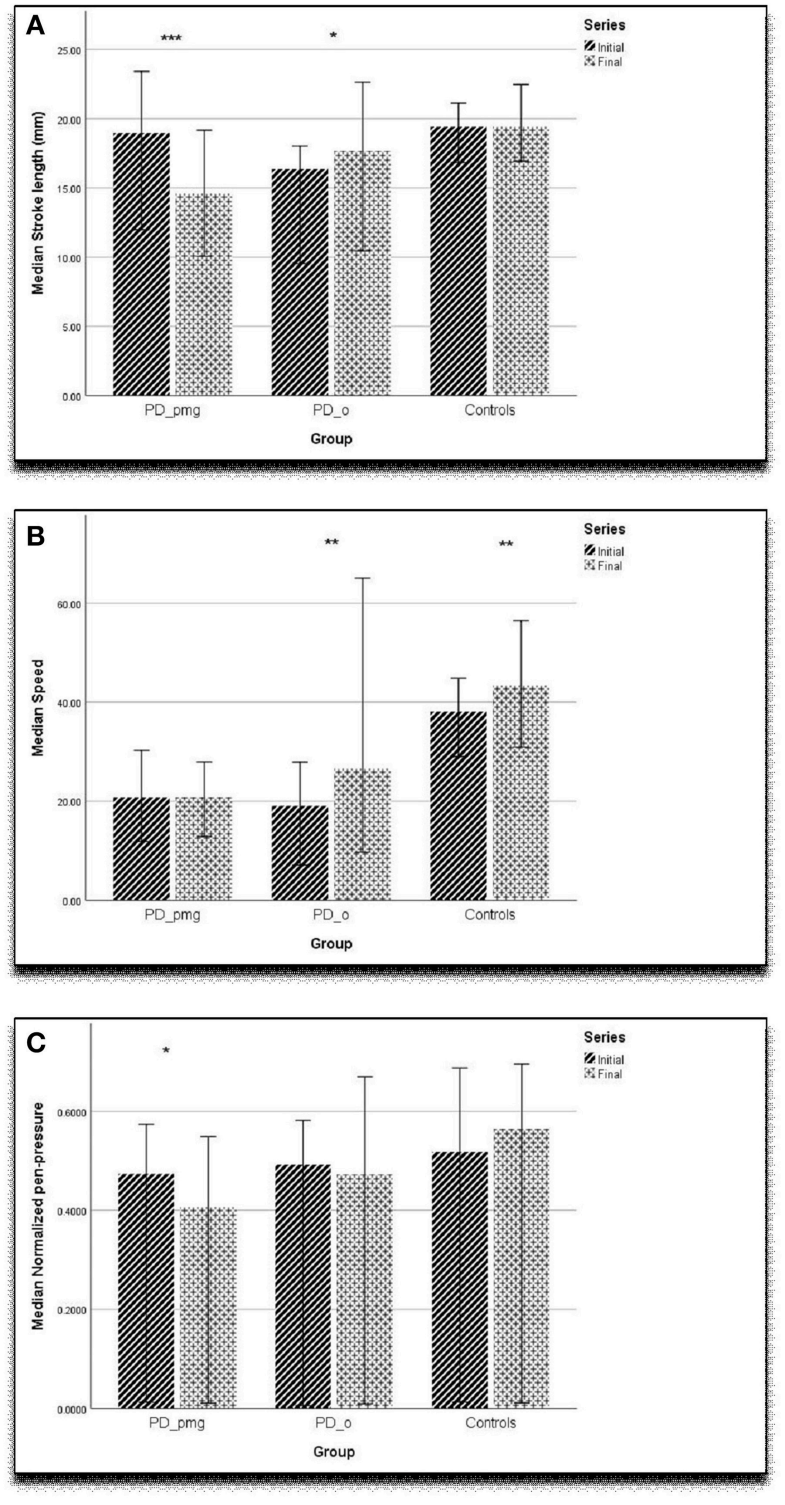
Graph showing **(A)** stroke length (mm), **(B)** speed (mm/sec), **(C)** normalized pen-tip pressure with error bar of 95% confidence interval. ***p < 0.001, **p < 0.01, *p < 0.05.

The “bradykinesia” of PD is a shorthand for complex disturbances of initiation and execution of actions and the ability to sustain them (30). Akinesia, a failure to initiate movement, and hypokinesia, describing underactive movement, are both related to bradykinesia, as is the sequence effect—repetitive movements becoming smaller or slower (31, 32). A closer examination of our results reveals more about the relationships between bradykinesia and PMG. In PMG, the decrement in writing size was accompanied by the normal decrease in vertical amplitude. Although this group had lost the normal horizontal kinematic augmentation of acceleration with left-to-right writing, speed was not decreased. Pressure measurements show that writing force is also impaired perpendicular to the writing plane in PMG. Both controls and PD_o subjects maintained writing pressure across the writing task. The PD_pmg subjects showed a significant decrement in pen-pressure between the initial and final letter series (Figure 2C). Together, the reduced acceleration and pressure measurements suggest that PMG reflects poorly sustained net force.

While the decrement in writing amplitude and force in PMG closely resembles the sequence effect of general bradykinesia, we did not find any significant correlation with overall *off* state parkinsonian motor disability, nor with the aggregate UPDRS-III bradykinesia scores for the dominant arm. These scores were similar for parkinsonian subjects with and without PMG. One possible reason is that, while micrographia and bradykinesia are related, there are fundamental task-related differences. Functional MR images described by Wu et al. (5) suggested that, in addition to dysfunctional basal ganglia motor circuits, PMG was associated with disconnections between the rostral supplementary motor area, rostral cingulate and motor area, and cerebellum.

Based on stroke length, 4 out of 24 PD participants fell below 2 standard deviations of control values and thus fulfilled criteria for consistent micrographia proposed by Kim et al. (8). However, only one of these patients had a purely consistent pattern, while the other 3 also had progressive micrographia. Using the quadrilateral letter area method of Ma et al. (14), none of our parkinsonian subjects had consistent micrographia. Thus, our findings cast doubt on the usefulness of subdividing parkinsonian micrographia into consistent and progressive categories, at least according to the definition of Kim et al. (8). A caveat is that our research investigated Roman script, while Korean and Chinese characters, which comprise multiple distinct strokes, were used in the studies just cited. Whether a definition of consistent micrographia based on specimens of premorbid calligraphy would work better is not clear. One difficulty would be the establishment of a “typical” premorbid script size, since the size of handwriting in normal subjects is itself dependent on various factors such as speed and urgency of writing, writing implement, writing surface and scale of writing paper, including ruled lines (33).

Our findings agree with earlier studies that kinematic measures of acceleration and speed (Figure 2B) are slower in PD when compared with controls (4, 9). As has been previously proposed, computerized kinematic analysis of handwriting may be sensitive enough to detect the earliest motor manifestations of PD in at-risk subjects (9). Since, PMG is only present in two-thirds of parkinsonian patients, decrement of writing amplitude might not be a reliable early discriminator (Figure 2A). Our work indicates that horizontal acceleration profile in left-to-right writing and pen pressure measurements are likely to be important in detecting subtle PMG when it is present.

A number of study limitations should be acknowledged. Our sample size is smaller than used in some previous handwriting research, though it was based on power calculations and proved adequate to reveal significant group differences. We took the view that OFF states were likely to reveal more about the PMG phenomenon, and we did not report the effect of levodopa medication. Ling et al. (7) and Wu et al. (5) did not find any significant improvements of writing decrement in ON states. We used the change in size between initial and final 5 letters to identify PMG which was to reduce the inter-experimental variability. An alternative approach, regression analysis of the whole writing task, has been employed, in different ways, by other researchers. We observed variations in the size of the characters during sustained handwriting. Many participants would hesitate briefly when writing to adjust letter size, resulting in several cycles of decrement rather than a steady, linear decline. We concluded that regression analysis of decrement was less suitable for our writing task.

Our reasons for adopting an absolute rather than probabilistic definition for PMG are presented in Introduction. Four control participants (16.7%) also met the definition for PMG. This is in line with recent research on healthy older subjects and should not be taken as evidence that our PMG criterion was insufficiently stringent. Among 185 individuals with slightly younger mean age than our control group, 21% had slowness of repetitive finger movement and 18% met a definition for mild parkinsonism (34). Single character tasks, word copying and free writing have all been used before to study PMG. We favored a single character task because this gave the best standardization for the kinematic comparisons and reduced compounding factors such as cognitive loading which has been shown to affect the kinematics of writing (35, 36). The letter *e* is well-suited to differentiating horizontal and vertical movements. Mean pen speeds were somewhat slower than some previous studies, though comparable to others (37). Most participants adopted a cursive writing style, yet they were required to separate rather than conjoin letters. A degree of deliberateness may have affected writing speed.

Previous studies of PMG have concentrated largely on dimensional aspects of writing, and our kinematic analysis contributes new knowledge about its dynamic characteristics. We add to understanding of the interplay between “horizontal micrographia” and progressive change (38). Recent research by Tinaz et al. (39), using isometric repetitive handgrip, associated the sequence effect in PD with motor energetic deficiency. Our findings on the shortfalls of force and acceleration in PMG suggest a similar problem with the transfer of energy into muscular movement and sustained contraction. Despite the lack of correlations with UPDRS-III scores, bradykinetic motor decrement and PMG appear to reflect a common defect with the energy efficiency of motor programmes.

## Ethics Statement

The study was conducted in accordance with the Helsinki Declaration on human experiments (revised 2004) and approved by the Monash Health and RMIT University Human Research Ethics Committees. All participants in this study gave their written informed consent prior to data recording.

## Author Contributions

PZ involved in conducting experiments, data analysis, drafting the article, software design and development, selection of analytical tools, and literature review. DK involved in concept and design of work, selection of analytical tools, critical revision of the article, literature review, participated in manuscript preparation, and final approval of the version to be published. PK involved in clinical support, critical revision of the article, and participated in manuscript preparation. SP involved in statistical analysis, and review of the article. KW and KN involved in experimental support. SR involved in clinical support, access to patient details, and experimental design. All authors were involved in manuscript review.

### Conflict of Interest Statement

The authors declare that the research was conducted in the absence of any commercial or financial relationships that could be construed as a potential conflict of interest.
